# Remote Photoacoustic Sensing Using Single Speckle Analysis by an Ultra-Fast Four Quadrant Photo-Detector

**DOI:** 10.3390/s21062109

**Published:** 2021-03-17

**Authors:** Benjamin Lengenfelder, Martin Hohmann, Moritz Späth, Daniel Scherbaum, Manuel Weiß, Stefan J. Rupitsch, Michael Schmidt, Zeev Zalevsky, Florian Klämpfl

**Affiliations:** 1 Institute of Photonic Technologies, University Erlangen-Nürnberg (FAU), Konrad-Zuse-Straße 3/5, 91052 Erlangen, Germany; Martin.Hohmann@lpt.uni-erlangen.de (M.H.); Moritz.Spaeth@lpt.uni-erlangen.de (M.S.); danielscherbaum@gmail.com (D.S.); Michael.Schmidt@lpt.uni-erlangen.de (M.S.); Florian.Klaempfl@lpt.uni-erlangen.de (F.K.); 2 Erlangen Graduate School in Advanced Optical Technologies (SAOT), Paul-Gordan-Straße 6, 91052 Erlangen, Germany; zeev.zalevsky@biu.ac.il; 3 Sensor Technology, University Erlangen-Nürnberg (FAU), Paul-Gordan-Straße 3/5, 91052 Erlangen, Germany; manuel.weiss@fau.de; 4 Laboratory for Electrical Instrumentation and Embedded Systems, Department of Microsystems Engineering—IMTEK, University of Freiburg, Georges-Koehler-Allee 106, 79110 Freiburg, Germany; stefan.rupitsch@fau.de; 5 Faculty of Engineering, Bar-Ilan University, Ramat-Gan 52900, Israel

**Keywords:** photoacoustic, remote sensing, endoscopy, speckle

## Abstract

The need for tissue contact makes photoacoustic imaging not applicable for special medical applications like wound imaging, endoscopy, or laser surgery. An easy, stable, and contact-free sensing technique might thus help to broaden the applications of the medical imaging modality. In this work, it is demonstrated for the first time that remote photoacoustic sensing by speckle analysis can be performed in the MHz sampling range by tracking a single speckle using a four quadrant photo-detector. A single speckle, which is created by self-interference of surface back-reflection, is temporally analyzed using this photo-detector. Phantoms and skin samples are measured in transmission and reflection mode. The potential for miniaturization for endoscopic application is demonstrated by fiber bundle measurements. In addition, sensing parameters are discussed. Photoacoustic sensing in the MHz sampling range by single speckle analysis with the four quadrant detector is successfully demonstrated. Furthermore, the endoscopic applicability is proven, and the sensing parameters are convenient for photoacoustic sensing. It can be concluded that a single speckle contains all the relevant information for remote photoacoustic signal detection. Single speckle sensing is therefore an easy, robust, contact-free photoacoustic detection technique and holds the potential for economical, ultra-fast photoacoustic sensing. The new detection technique might thus help to broaden the field of photoacoustic imaging applications in the future.

## 1. Introduction

Photoacoustic imaging (PA) is a new, rising imaging technique since it combines a high penetration depth with a good image contrast [1]. As PA is based on the absorption of a short light pulse and the subsequent acoustic signal generation, the absorption characteristics define the imaging contrast. As the absorption coefficients for different tissue absorbers differ greatly, it is possible to obtain a high image contrast. Hence, PA is especially attractive for displaying blood vessels due to the high hemoglobin absorption compared to other tissue constituents in the visible and near-infrared range [2]. This high contrast is combined with a high penetration depth of several millimeters since the acoustic scattering is up to a factor of 1000 less than the optical scattering [3]. Thus, higher imaging depths than for purely optical methods can be achieved with ease.

Since piezo element transducers, which are contact based, are the state-of-the-art detection technique, PA requires tissue contact. As a consequence, PA may not be suitable for special medical applications, such as wound imaging or laser surgery. Furthermore, transducer miniaturization is challenging, and small elements suffer from low sensitivity, which limits their usage for photoacoustic endoscopy. Therefore, a remote and stable detection system would be beneficial.

Air-coupled transducers could overcome this issue. However, they suffer from poor sensitivity and are still too big for minimally-invasive medical applications [4]. For the non-contact beam deflection technique, the deflection of a probe beam that is positioned above the tissue is monitored by a position sensitive detector [5,6]. A small fraction of the photoacoustic signal is transmitted to the surrounding air and deflects the probe beam. Since the probe beam needs to be scanned above the whole tissue surface, this modality is unsuitable for surgical usage. In addition, the detection bandwidth is limited due to the probe beam size. Interferometric detection methods offer non-contact sensing and a higher detection bandwidth. Here, the surface displacement after photoacoustic signal generation is monitored for the initial pressure reconstruction inside the object [7,8,9,10]. Nevertheless, interferometric systems are expensive and require a complicated setup that is noise sensitive [11]. In addition, interferometric setups only monitor the surface displacement correctly, if the surface is properly tilted, which cannot be guaranteed for clinical application. There are also non-interferometric approaches for remote acoustic signal detection. Clark et al. detected surface acoustic waves by tracking the movement of multiple speckles using simple diode detection systems [12,13]. For this approach, however, the speckles are imaged in the near-field, and thus, Fresnel diffraction applies. In this case, the movement of the speckles is dependent on three types of movement, which cannot be separated and occur simultaneously: transverse, axial, and tilt [14]. Due to this disadvantage, the acoustic sensing approach of Clark et al. may not be suitable for photoacoustic imaging. Hajireza et al. sensed the photoacoustic signal directly at its origin by a non-interferometric system [15]. This system monitors the reflection of a probe beam that is sensitive to the elasto-optic index modulation induced by the photoacoustic initial pressure transients. This approach allows non-interferometric, non-contact photoacoustic sensing. However, it provides only penetration depths in the mm-range due to the high optical attenuation for the probe beam. In addition, it is only applicable for photoacoustic microscopy and not suited for photoacoustic tomography since it does not provide temporally resolved acquisition of the acoustic signal and thus requires depth scanning for image acquisition.

Remote speckle analysis is an easy, robust, and non-interferometric vibration sensing technique whose potential for photoacoustic tomography we already demonstrated in previous publications [16,17,18]. By tracking the movement of multiple speckles, it was possible to remotely reconstruct the photoacoustic signal. However, in these proof-of-concept studies, the acoustic sensing bandwidth and thus resolution were limited to the frame rate of the expensive, high-resolution camera used at approximately 800 kHz.

In the present publication, it is demonstrated for the first time that remote photoacoustic sensing with a sampling rate of 8 MHz can be performed by tracking the movement of a single speckle with only four diodes. The speckle tracking by only four diodes proves the applicability of an economical position sensitive diode for speckle analysis. Polymer phantoms and skin tissue samples are measured in transmission mode and reflection mode. In addition, the capability of easy miniaturization and endoscopic usage of the single speckle analysis is shown by fiber bundle measurements. The new technique is an essential step for the implementation of a remote photoacoustic imaging system using speckle analysis. Therefore, this work might help to broaden the applications of PA in special applications like wound imaging, endoscopy, or guiding laser surgeries.

## 2. Materials and Methods

### 2.1. Speckle Sensing by Multiple Speckles’ Tracking

**Figure 1:**
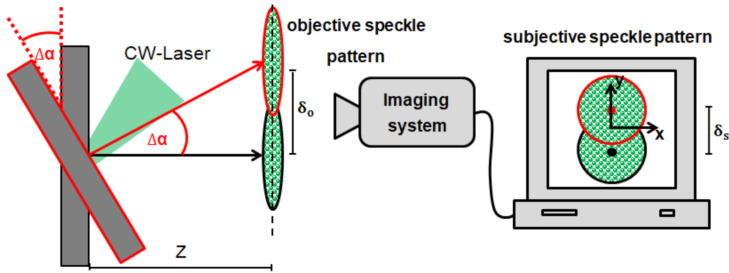
Speckle sensing theory: An object tilt results in a speckle pattern movement, which can be reconstructed with an imaging system. Figure after [19].

The speckle sensing technique is based on the time-resolved detection of the position of a speckle pattern. It is possible to extract the tilt change of a laser illuminated surface by tracking the speckle pattern movement [14]. The left side of Figure 1 shows a tilting object surface, and the right side illustrates the speckle sensing technique.

If the observation plane distance *Z* for the generated objective speckle pattern fulfills the far-field approximation (*Z* > ($D_{ill}^{2}$)/(4λ), illuminated diameter $D_{ill}$), the objective speckle pattern movement ($\delta_{o}$) is only dependent on the surface tilt change Δα and *Z*. By imaging the objective speckle pattern using an imaging system with the magnification *M*, a subjective speckle pattern is created on the imaging sensor whose lateral movement ($\delta_{s}$) is also linearly proportional to Δα assuming small angle approximation. Equation (1) explains the relation between the speckle movements, *M* and *Z* [19].

(1) $$\delta_{s}=\delta_{o}\cdot M=tan\left(\Delta \alpha \right)\cdot Z\cdot M$$

By acquiring a speckle pattern video sequence with the imaging system, it is thus possible to measure the surface tilt. Correlation analysis of the image frames allows the reconstruction of the speckle pattern movement along the sensor axes x and y, which are representative of the surface tilts along these two axes’ directions [19]. In this article, it is shown for the first time that speckle sensing is feasible by tracking the movement of a single speckle in contrast to the described correlation analysis of a multiple speckle image. Therefore, it is proven that a single speckle contains all necessary information for photoacoustic signal detection.

### 2.2. Imaging Systems and Setups

Two imaging systems are established for this work and described here in detail: the free-space setup, which is capable of far distance PA sensing, and the fiber based setup, which is suitable for endoscopy.

#### 2.2.1. Free-Space Single Speckle Sensing

**Figure 2:**
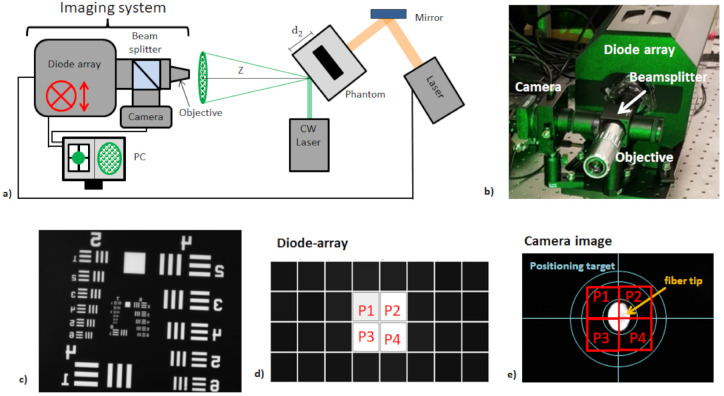
(**a**) Experimental setup (transmission mode) for the remote photoacoustic measurements using the diode array sensing system. The imaging system can be moved mechanically in lateral directions, which is indicated by the red marks. (**b**) Picture of the sensing system. (**c**) The magnification of the sensing system is calculated at 5 using a USAF 1951 Test Target. (**d**,**e**) The camera position of the sensing system is calibrated using a multi-mode fiber. The diode signals and the camera image are shown when an illuminated fiber is placed in their center. Figures after [20].

Figure 2 shows the established diode based, free-space imaging system. The imaging system consists of a microscope objective (10×), a beamsplitter and a lens (*f* = 100 mm) used for image magnification on a camera (DCC1545M, Thorlabs, Newton, NJ, USA), and an avalanche-photodiode sensor (APS; APDcam, Fusion Instruments, Budapest, Hungary). A bandpass filter for the speckle wavelength is placed after the objective in order to block the photoacoustic excitation and room light. The magnification of the system is calculated at 5 with a microscope test target (*M* = 5). The camera is used as a reference for image calibration and alignment. The calibration is done with a multi-mode fiber (AFS105/125Y, Thorlabs, Newton, NJ, USA) to which a halogen light source (HL-2000, Ocean Optics, Ostfildern, Germany) is coupled. The fiber tip is then imaged on the camera and APS (Figure 2e), and the co-alignment of the camera image and the APS can be verified. The APS consists of a 4×8 avalanche-diode array (pixel size: 1.6 mm) and therefore offers 32 pixels. Of these 32 pixels, only the four central pixels are used for the tracking of a single speckle in order to maximize the acquisition rate.

**Figure 3:**
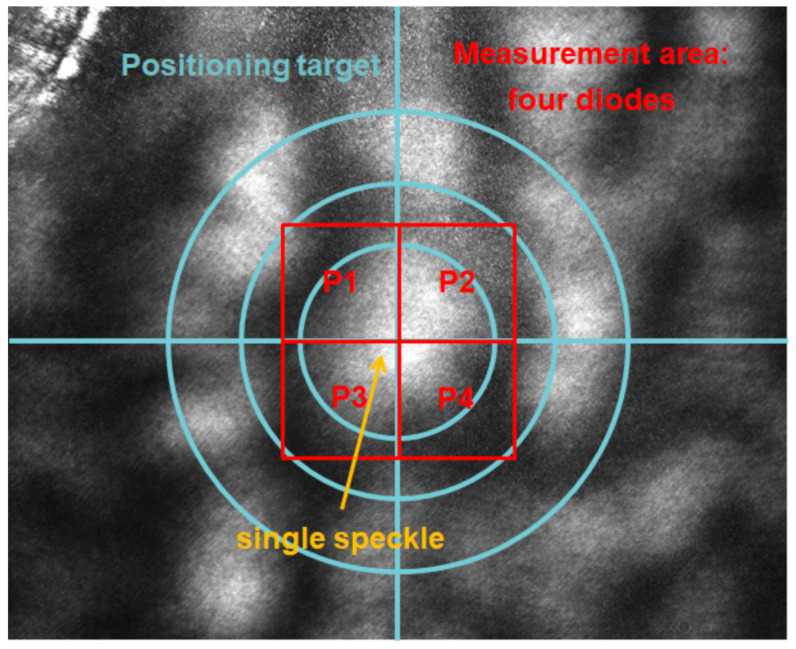
The camera image of an exemplary speckle used for photoacoustic measurement is shown. The speckle is placed in the center of the reference camera (blue target) and thus in the center of four measurements diodes P1/P2/P3/P4 (red) by manually moving the complete imaging system.

The speckles are generated by cw illumination (532 nm, 80 mW, diameter: 0.75 mm) of the sample surface and imaged at *Z* = 20 cm. First, a convenient speckle is found by manually moving the imaging system using mechanical stages and visually tracking the camera image. A speckle is considered as convenient for the measurement if it is in the center of the camera and therefore in the center of the diode array (see Figure 3). Furthermore, the speckle size needs to be in the range of the pixel size of the diode array (1.6 mm). This is ensured by comparing the tracked speckle size to the inner circle (diameter 1.4 mm) of the illustrated target in Figure 3, which can be displayed by the camera software.

For the photoacoustic measurements, the samples are excited with a short laser pulse (Q-Smart 450, Quantel laser, Les Ulis, France). The laser parameters are as follows: λ = 1064 nm, pulse duration 5 ns, beam diameter 7 mm, pulse energy 90 mJ. These parameters result in an exposure of 230 $\frac{\mathrm{mJ}}{{\mathrm{cm}}^{2}}$, which is above the maximum permitted exposure (MPE) for single pulse excitation at 1064 nm (100 $\;\frac{J}{{\mathrm{cm}}^{2}}$). This, however, is desired to achieve a high signal amplitude for the demonstrated proof-of-concept experiments. The laser pulse triggers the acquisition start of the imaging system with a sampling rate of 8 MHz.

In order to prove the safe applicability of the sensing system, ex vivo skin measurements are performed with a total exposure below the MPE. This is achieved by replacing the described cw laser with a temporally pulsed laser diode (IBEAM-SMART-405-S-HP, Toptica Photonics, Gräfelfing, Germany) and by reducing the photoacoustic excitation energy. The laser diode pulse (λ = 405 nm) is temporally triggered by the short laser pulse for photoacoustic excitation and illuminates the tissue only for a duration of 30 μs after photoacoustic excitation at a peak power of 35 mW with an illumination radius of 300 μm at the tissue surface. This temporal speckle illumination together with a reduced excitation energy of 35 mW results in a total exposure that is below the MPE for soft tissue [21].

#### 2.2.2. Fiber-Based Single Speckle Sensing

**Figure 4:**
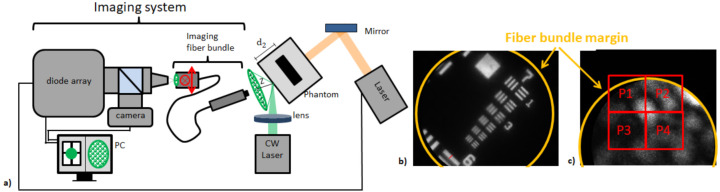
Experimental setup and imaging unit for the remote photoacoustic measurements using the fiber based approach (**a**). The proximal fiber bundle end can be moved in lateral directions, which is indicated by the red marks. The magnification for the camera arm is determined at 50 using a USAF 1951 Test Target (**b**). A convenient speckle that is centralized inside the photodiode measurement area is illustrated (**c**).

Figure 4 shows the imaging unit and setup for the fiber based approach and camera images of a USAF 1951 Test Target and a selected speckle.

In contrast to the free-space approach, an imaging fiber bundle (30,000 fibers, imaging resolution 1 μm, working distance 30 μm, field of view diameter 240 μm) that can be used in endoscopy is used for speckle pattern imaging. At the proximal fiber bundle end, the speckles are imaged by the diode based imaging system mentioned in Section 2.2.1. In contrast to the free-space system previously described, a 20× magnification objective (WC95248318, Mitutoyo, Japan, 20×) is used. Furthermore, the imaging lens in the diode array arm is changed to *f* = 200 mm. Together with the magnification of the imaging fiber bundle (M=2.5), these changes result in a magnification of M=50 for the diode array arm and M=25 for the camera arm. These higher optical magnifications allow speckle sensing at a near imaging distance of *Z* = 2 mm, which is desired for endoscopic usage.

The speckles are generated by focused cw illumination (532 nm, 100 mW, $D_{ill}=50\;$ μm) of the sample surface and imaged by the fiber bundle and APS system at Z=2 mm. A convenient speckle is found and automatically centered inside the measurement area of the four photodiodes by analyzing the camera image and moving the proximal fiber bundle end in lateral directions. For the photoacoustic measurements, the phantoms are excited with a short laser pulse (Q-Smart 450, Quantel laser, Les Ulis, France), and the laser parameters are the following: λ=1064 nm, pulse duration 5 ns, beam radius 7 mm, pulse energy 110 mJ. These parameters result in an exposure dose of 285$\;\frac{\mathrm{mJ}}{{\mathrm{cm}}^{2}}$, which is above the MPE for single pulse excitation at 1064 nm (100$\;\frac{J}{{\mathrm{cm}}^{2}}$). This, however, is desired to achieve a high signal amplitude for the proof-of-concept experiments. The laser pulse triggers the acquisition start of the APS with a sampling rate of 8 MHz.

### 2.3. Measurement Modes and Samples

Using the described free-space and fiber based imaging systems, the samples are measured in transmission mode and reflection mode. For transmission mode, the photoacoustic excitation and speckle sensing take place at opposite sample sides, whereas they are on the same side for reflection mode.

The phantoms used in this work are made of the soft polymer PVCP (polyvinyl chloride plastisol; Standard Lure flex (medium), Lure Factors, Great Britain) and consist of two parts: absorber and scattering matrix. In order to adjust the optical properties for these parts, additives are used during the plastisol preparation process. A black plastic color changes the absorption coefficient $\mu_{a}$, and TiO${}_{2}$-particles (titanium(IV)-oxide, Sigma Aldrich, Taufkirchen, Germany) adjust the reduced scattering coefficient $\mu_{s}^{\prime }$. In this work, a color concentration of 7-vol-% and a TiO${}_{2}$-concentration of $4\;\frac{\mathrm{mg}}{\mathrm{mL}(\mathrm{PVCP})}$ is used for the absorbing and scattering phantom parts, respectively. The optical properties for these concentrations were determined at the excitation wavelength 1064 nm using spectrophotometric measurements and inverse adding doubling. The absorption coefficient for the absorbing phantom part is 106 cm${}^{-1}$, and the reduced scattering coefficient for the scattering part is 21 cm${}^{-1}$. The scattering coefficient for the absorbing part and the absorbing coefficient for the scattering part can be neglected.

For the measurements using the free-space setup, three different PVCP phantoms (PhAT1, PhAT2, PhAT3) with increasing distances between the absorber surface and detection surface (d) are measured in transmission mode. The “Ph” in the phantom name stands for phantom, “A” for the free-space setup, and “T” for transmission mode. Two PVCP phantoms are measured in reflection mode (PhAR4, PhAR5). The “R” in the sample name stands for reflection mode. Two skin tissue samples (skinAT1, skinAT2p) are measured in transmission mode. The “p” in the sample name stands for the pulsed speckle illumination, which ensures a total exposure below the MPE for the experiments with the skin tissue. The skin tissue was obtained from bisected pig heads, which were obtained from the local slaughterhouse (Unifleisch GmbH, Erlangen, Germany). Therefore, the approval of the Ethics Committee was not necessary. The tissue sample was prepared manually using a scalpel. For skinAT1, a PVCP-absorber was placed at the sample bottom by cutting out a hole in the tissue. For skinAT2, a PVCP-absorber was placed between a fat layer, which was obtained from a local supermarket, and a skin layer. In order to ensure good contact between the sample constituents, ultrasound gel was used, and for skinAT2p, a metallic sample holder gently pressed the tissues.

The speed of sound for the prepared skin sample was measured at 1300$\;\frac{m}{s}$ and for the PVCP phantoms at 1330$\;\frac{m}{s}$ with an ultrasound thickness measurement device (Mini Test 430, Elektro Physik, Germany). For each sample measured with the free-space setup, fifteen measurements were analyzed in order to ensure statistical relevance.

For the measurements using the fiber based imaging setup, two PVCP phantoms (PhBT1, PhBT2) were measured in transmission mode. The “B” in the sample name stands for the fiber based setup. The speed of sound for these samples were measured at 1349$\;\frac{m}{s}$ with an ultrasound thickness measurement device (Mini Test 430, Elektro Physik, Germany). For these two phantoms, ten measurements were analyzed in order to ensure statistical relevance.

The density ρ of all PVCP phantoms was measured by the volume displacement of ethanol at 1200$\;\frac{\mathrm{kg}}{m^{2}s}$. The resulting acoustic impedance ($Z_{ac}=\rho c$) of the used phantoms in this work was therefore in the range of $1.60\times 10^{6}\frac{\mathrm{kg}}{m^{2}s}$–$1.62\times 10^{6}\frac{\mathrm{kg}}{m^{2}s}$, which is in good agreement with the values of soft tissue: the impedance of fat tissue is $1.4\times 10^{6}\frac{\mathrm{kg}}{m^{2}s}$ and for muscle $1.62\times 10^{6}\frac{\mathrm{kg}}{m^{2}s}$ [22].

**Figure 5:**
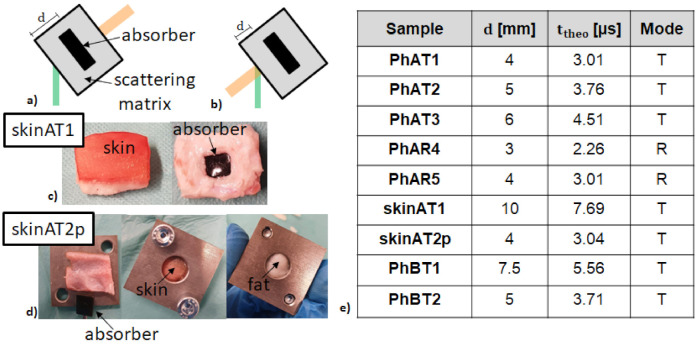
The transmission setup (T) is sketched, and the phantom parts are marked (**a**). The reflection setup (R) is illustrated (**b**). Pictures of the two skin tissue samples are shown: skinAT1 with an absorber at the bottom (**c**) and skinAT2p with an absorber between skin and fat (**d**). The corresponding detection distances, theoretical detection times, and measurement modes are summarized in the table (**e**).

Figure 5 sketches the phantom position in regard to the excitation and illumination laser for the two measurement modes and shows the corresponding detection distances d of all samples. The absorber thickness is 3 mm for all samples, and d is varied by adjusting the thickness for the scattering sample part. For all samples, the mean detection times by automated single speckle analysis and their standard deviations are compared to the theoretical detection time.

### 2.4. Data Analysis for Single Speckle Analysis

**Figure 6:**
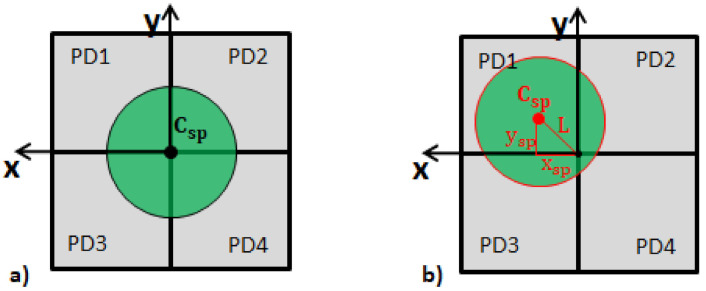
A perfectly round speckle, which is initially in the center of the measurement diodes is shown (**a**). The diode signals (P1, P2, P3, P4) can be used to compute its center of gravity $C_{sp}\left(x_{sp},y_{sp}\right)$ and its total vector length *L* in order to detect speckle movements (**b**).

Figure 6 shows a speckle, which is initially in the center of the four measurement diodes P1, P2, P3, and P4 and moves to a different position due to the PA signal.

By using the temporal signals of the four diodes, it is possible to compute the temporal center of gravity of the speckle $C_{sp}$ ($x_{sp}$, $y_{sp}$) and its total vector length *L* by using Equations (2)–(4), similar to a four quadrant position sensitive diode [23]. Since the speckle moves if the surface tilts, this center of gravity is related to the photoacoustic signal, and it is possible to reconstruct the absorber depth *d*.

(2) $$x_{sp}=\frac{\left(P1+P3)-(P2+P4\right)}{\left(P1+P2+P3+P4\right)}$$

(3) $$y_{sp}=\frac{\left(P1+P2)-(P3+P4\right)}{\left(P1+P2+P3+P4\right)}$$

(4) $$L=\sqrt{x_{sp}^{2}+y_{sp}^{2}}$$

In order to prove the usability of the sensing system for remote photoacoustic detection, the detection times of $x_{sp}$, $y_{sp}$, and *L* are shown for the free-space approach. For *L*, the mean detection time $t_{mean}$ and its standard deviation σ are computed for all samples and verified to $t_{theo}$ for the acoustic signal, which is calculated using d and c.

### 2.5. Sensing Parameter Evaluation for Single Speckle Analysis

#### 2.5.1. Sensitivity

The sensitivity in terms of minimal detectable tilt $S_{d,\alpha }$ is limited by the noise floor $\sigma_{nf}$ of the PA measurements. The noise level $\sigma_{nf}$ is computed by taking the standard deviation of a PA measurement data set before excitation. For this standard deviation computation, one-hundred fifty data points before PA excitation are used. By determining the noise floor $\sigma_{nf}$ and taking into account the relevant imaging parameters (*Z*, pixel size $d_{px}$, magnification *M*, illumination diameter $D_{ill}$), $S_{d,\alpha }$ can be determined. The minimal detectable speckle shift $\delta_{s,min}$ is equal to the multiplication of $d_{px}$ and $\sigma_{nf}$. By considering Equation (1), it is then possible to compute the minimal detectable tilt $S_{d,\alpha }$: $\tan\left(S_{d,\alpha }\right)=\frac{\delta_{s,min}}{ZM}$. Under the assumption that the investigated speckle pattern or single speckle consists of reflections from the complete illuminated surface area with the diameter $D_{ill}$, the minimal detectable axial surface deformation is estimated by $S_{d,nm}=\tan\left(S_{d,\alpha }\right)\cdot D_{ill}$. The minimal detectable pressure $S_{d}$ can be determined according to Equation (5) [24].

(5) $$S_{d}=\pi Z_{ac}S_{d,nm}f$$

The parameter *f* defines the frequency of the acoustic wave that should be detected, and $Z_{ac}$ is the acoustic impedance. For the established system here, the maximum detectable frequency is half the frame rate. For the established free-space and fiber based sensing system, the described sensitivities are computed and compared to the literature and to contact ultrasound transducers.

#### 2.5.2. Sensing Range

The sensing range, i.e., the tilt interval that is covered by the remote speckle analysis, is dependent on the sensitivity, speckle size, and sensor size. The sensitivity defines the minimal detectable tilt as treated in the previous section. The maximal detectable tilt $\alpha_{max}$ is defined by the sensor and speckle size. The sensing range for the single speckle analysis is discussed.

#### 2.5.3. Linearity

**Figure 7:**
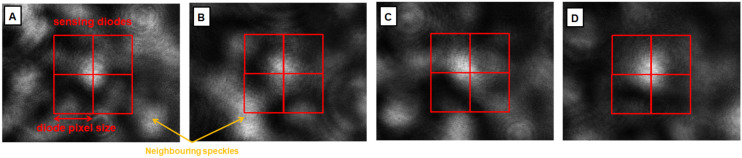
Speckle images (**A**–**D**) that are evaluated for their single speckle sensing capability. The diode sensing regions are indicated by the red rectangles.

Simulations were carried out in order to evaluate the linearity and robustness against neighboring speckles, meaning speckles that are not situated inside the original image, but appear in the shifted speckle image. Four speckle patterns with a centralized speckle were analyzed. Figure 7 shows these speckle patterns. The images are moved in the horizontal direction with a shift amplitude of −0.26 to 0.26 ($x_{sp,real}$) of the diode pixel size with a shift resolution of 0.025. For each shifted image, the horizontal center of gravity $x_{sp}$ for the fixed sensing diodes region, which is indicated in Figure 7, is computed according to Equation (2). The values of $x_{sp}$ are computed for all shifts and speckle images, compared to $x_{sp,real}$ and compared to linear behavior.

## 3. Results and Discussion

### 3.1. Photoacoustic Measurements

**Figure 8:**
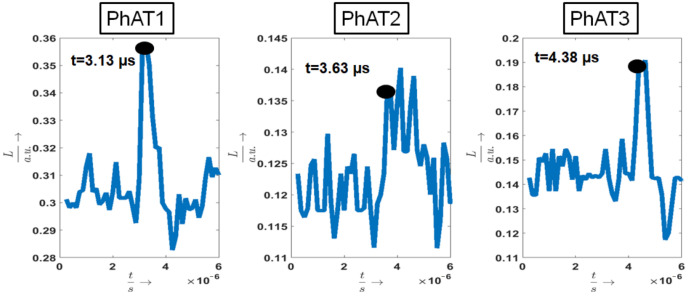
The speckle vector length $t_{sp}$ is illustrated for measurements of phantoms PhAT1–PhAT3. For the three samples, the detection times of the initial generated photoacoustic signal are noted, and the corresponding signal peaks are marked.

Figure 8 shows measurement results in transmission mode of the three phantoms PhAT1, PhAT2, and PhAT3. They illustrate the speckle vector length *L*, which represents the temporal vibration profile of the surface under investigation. The detection time of the first peak in these profiles is marked with a black circle, because this time point corresponds to the photoacoustic signal. The surface expansion after the photoacoustic excitation results in a surface tilt change and thus in a speckle movement, which can be seen in *L*. For the phantoms PhAT1-PhAT3, the acquisition times increase as expected with increasing acoustic travel distance *d*. Considering $t_{sp}$, the acquisition time increases as follows: 3.13 μs, 3.63 μs, and 4.38 μs. By using the speed of sound (1330$\;\frac{m}{s}$), the following acoustic travel distances are calculated: 4.163 mm, 4.828 mm, and 5.825 mm. Thus, for each phantom, the acquisition time of the photoacoustic signal by speckle analysis corresponds to the geometrical phantom dimensions (d: 4 mm, 5 mm, 6 mm), taking into account the phantom production uncertainty.

**Figure 9:**
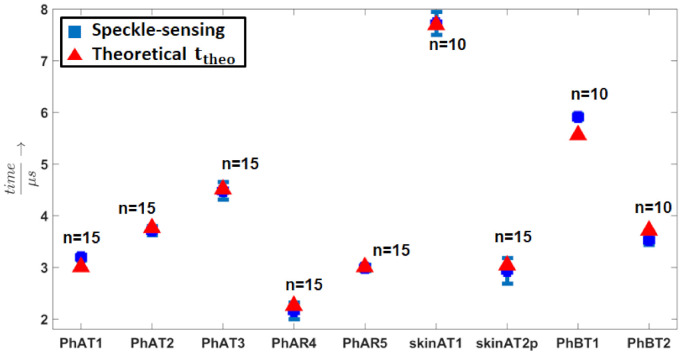
Mean detection times ($t_{mean}$) and their standard deviation σ for the photoacoustic measurements. The sample names can be explained as follows: Ph stands for a phantom, A for the free-space setup, B for the fiber based setup, T for transmission mode, R for reflection mode, and p for pulsed speckle illumination below the MPE. The theoretical transit time of the photoacoustic signal is used for verification.

Table 1 summarizes $t_{mean}$ for all photoacoustic acquisition times of all samples considering *L*, their σ, and the theoretical acoustic transit time $t_{theo}$. For illustration purpose, these results are also plotted in Figure 9.

It is clearly visible that the speckle analysis mean detection times increase with bigger phantom dimensions and that they match the acoustic transit times. The small differences between the mean and theoretical detection time can be explained with inaccuracies for the phantom manufacturing and measuring process. The low standard deviations prove the repeatability and the fact that single speckle sensing allows precise photoacoustic sensing compared to the previous high-speed camera experiments. The standard deviation is in the range of approximately 0.1 μs, which results in a precision of 0.13 mm considering the speed of sound.

Based on the repeatability and successful verification with the theoretical transit time $t_{theo}$ of the transmission mode and reflection mode measurements, it can be concluded that free-space and fiber based single speckle sensing is a reliable technique for the photoacoustic detection on phantoms and on skin tissue samples. Furthermore, the less expensive low-resolution diode sensor, in contrast to the previously used high-speed camera, reaches a high sampling rate of 8 MHz, which allows precise photoacoustic sensing.

### 3.2. Sensing Parameters

#### 3.2.1. Sensitivity

Table 2 summarizes the relevant parameters for the determination of $S_{d,nm}$ and $S_{d}$ for the established sensing systems. For the purpose of comparison, *f* is selected at 1 MHz and 4 MHz, which represents the maximal detectable acoustic frequency for the established sensing system in this work.

With the systems developed in this work, axial deformations of approximately 5 nm can be detected, which results in a pressure sensitivity of approximately 20 kPa for a 1 MHz acoustic wave. Horstmann et al. reached the following sensitivity parameters with a full-field speckle interferometry approach: $S_{d,\mathrm{nm}}=1\;$ nm and $S_{d,1\mathrm{MHz}}=1.5$ kPa with a sensing bandwidth of 80 MHz [10]. These values, however, were achieved for measurements on silicone, which has a lower impedance ($0.94\times 10^{6}\;\frac{\mathrm{kg}}{m^{2}s}$) than PVCP, which results in low $S_{d}$. Piezoelectric contact transducers that are especially designed and optimized for broadband PA detection achieve high sensitivities, which are dependent on the size and detection bandwidth. For a detection of acoustic frequencies in the range of 10 MHz to 50 MHz with an element size of 30$\;{\mathrm{mm}}^{2}$, $S_{d}$ can be estimated to lie between 1.5 Pa and 3.5 Pa [25]. Arrays have a smaller active area per detector element and thus lower sensitivity. An optimized ultrasonic line array can have a sensitivity of 110 Pa for a single element [26]. These sensitivities are better than the sensitivity for the established sensing system in this work. However, as explained in the Introduction, the interferometric setup is more complicated than the speckle analysis applied in the present investigation, and in comparison to the transducer, the single speckle analysis is contact-free. In addition, the speckle analysis sensitivity might even be improved by the usage of smaller photodiodes, tighter focusing of the cw illumination, or new data analysis techniques.

#### 3.2.2. Sensing Range

The single speckle analysis tracks the center of gravity of a single speckle that is positioned in the center of the four measurement diodes (Equations (2) and (3)). In general, the maximal detectable center of gravity coordinates ($x_{sp,max}$, $y_{sp,max}$) are defined by the single speckle diameter $l_{s}$ and the diode size ($d_{px}$) by Equation (6). For a larger speckle shift in regards to the sensor center, the single speckle would not be on the sensor completely, which leads to measurement errors. The maximum allowable speckle diameter is defined by twice the diode size. When assuming a speckle diameter of the pixel size, the maximum detectable center of gravity coordinates ($x_{sp,max}$, $y_{sp,max}$) are thus half the diode size.

(6) $$x/y_{sp,max}=\left(1-\frac{l_{s}}{2d_{px}}\right)d_{px}$$

Table 3 gives an overview of the maximal detectable tilts in the horizontal and vertical direction ($\alpha_{max,x}$, $\alpha_{max,y}$). For the single speckle analysis, $x/y_{sp,max}$ can be determined according to Equation (6) with $l_{s}=0.6d_{px}$.

The detectable tilt interval can be defined at [${55\times 10^{-5}}^{\circ };0.0642^{\circ }$] for the diode system. These intervals can be converted according to the computations from Table 2 to pressure interval [31.67 kPa; 3690 kPa] for a PVCP surface and a 1 MHz acoustic wave. These values result in a dynamic range factor for the pressure detection of approximately 116, which is convenient for PA sensing.

#### 3.2.3. Linearity

**Figure 10:**
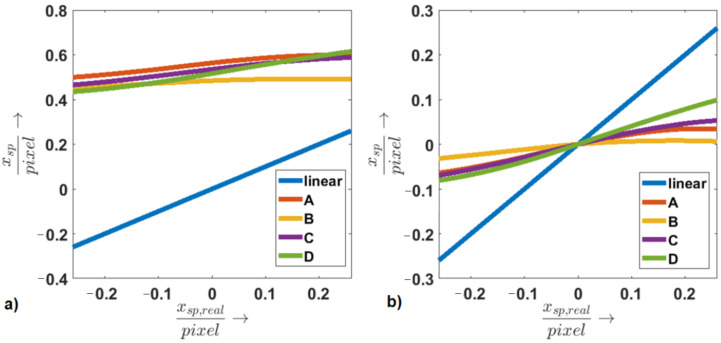
The computed values for $x_{sp}$ are shown over the $x_{sp,real}$ without (**a**) and with zero offset correction (**b**).

Figure 10 illustrates the computed value for $x_{sp}$ for all shifts and speckle images and compares the results to linear behavior.

Though we tried to centralize the speckle inside the camera image, $x_{sp}$ is not zero for a zero shift (see Figure 10a). This can be explained by the speckle surrounding signal, which also falls into the diode sensing regions. Due to this effect, there is an offset for the computed $x_{sp}$ compared to linear behavior. This offset can be corrected, although there is still a clear difference between the computed $x_{sp}$ and linear behavior (see Figure 10b), which depends on the central speckle size and intensity distribution. Furthermore, the connection between $x_{sp,real}$ and $x_{sp}$ might be surjective. This means that there can be multiple values of $x_{sp}$ for one $x_{sp,real}$. This effect occurs for Speckle Image B, as neighboring speckles that are shifted into the diode sensing region lower the signal strongly for higher shift magnitudes. The non-linearity problem can be corrected since each extracted shift can be mapped to a real shift when assuming a non-surjective behavior for the connection between $x_{sp}$ and $x_{sp,real}$. This could be assured by an automated speckle finding software that analyzes the camera image and potential values for $x_{sp,real}$ and identifies a suitable speckle automatically.

## 4. Conclusions and Outlook

In previous studies, we already demonstrated the feasibility of remote photoacoustic sensing by multiple speckles analysis [16,17,18,20]. However, in these previous works, expensive detector systems were used and multiple speckles were analyzed, which limits the achievable sensing rate and thus also the resolution. Furthermore, the detection systems used were not fiber based and thus not suitable for endoscopic applications.

This study reports on a new, purely optical, non-interferometric modality for PA signal acquisition in the MHz range by analyzing a single speckle with four diodes and demonstrates its suitability for endoscopy. Based on the repeatability and successful verification of the transmission mode and reflection mode measurements, it can be concluded that a single speckle provides the information required for reliable PA detection on phantoms that mimic the optical and mechanical properties of tissue and skin samples. The successful fiber based measurements demonstrate the usability of the approach for endoscopic applications. These results are essential steps toward the future application of the technique in a potential imaging device or as a smart feedback system for laser procedures.

However, several challenge arise for the implementation of a future imaging system. Higher sensing rates need to be achieved in order to provide better sensing resolutions. For this work, the frame rate was limited to 8 MHz by the APS, which could be replaced by an economical four quadrant position sensitive diode [27]. With appropriate hardware, this PSD allows higher acquisition rates and thus better sensing resolutions. An array of four quadrant diodes could be developed, and a measurement would be started if speckles were in a convenient position. Therefore, this sensor array would make a positioning unit obsolete and lower the costs of the sensing system significantly since a standard camera could be used for speckle position and shape tracking. Furthermore, the sensing sensitivity needs to be improved, which could be achieved by new hardware and a more precise speckle shift extraction algorithm [23]. This improved sensing sensitivity would reduce the required PA excitation exposure, which was above the MPE for soft tissue in most of the experiments for this investigation. A scan pattern of 10×10, which is considered to be sufficient for imaging, and a single photoacoustic measurement time of 10 μs result in a total measurement time of 1 ms per image, thus allowing imaging rates of 1 kHz. These mentioned steps together with appropriate reconstruction algorithms will allow PA imaging in the future [28].

## Figures and Tables

**Table 1:** The mean value $t_{mean}$ for all photoacoustic acquisition times of all samples considering, its standard deviation σ, and the theoretical acoustic transit time $t_{theo}$ are listed.

Sample	$t_{\mathrm{mean}}$ (μs)	σ (μs)	$t_{\mathrm{theo}}$ (μs)
PhAT1	3.19	0.06	3.01
PhAT2	3.71	0.09	3.76
PhAT3	4.48	0.17	4.51
PhAR4	2.15	0.16	2.26
PhAR5	2.99	0.06	3.01
skinAT1	7.72	0.22	7.69
skinAT2p	2.93	0.25	3.04
PhBT1	5.91	0.057	5.56
PhBT2	3.53	0.093	3.71

**Table 2:** Relevant parameters for the determination of $S_{d,nm}$ and $S_{d}$ for the established sensing systems.

	Free-Space		Fiber-Guided
	PVCP	skin	PVCP
$\sigma_{nf}$	0.006	0.007	0.0057
$d_{px}$ in μm	1600	1600	1600
*M*	5	5	50
*Z* in cm	20	20	0.2
$S_{d,\alpha }$ in ${10^{-5}}^{\circ }$	55.0	64.2	522
$D_{ill}$ in mm	0.75	0.75	0.05
$S_{d,nm}$ in nm	7.2	8.4	4.56
$Z_{ac}$ in $10^{6}\frac{\mathrm{kg}}{m^{2}s}$	1.4	1.99	1.4
$S_{d,1\;\mathrm{MHz}}$ in kPa	31.67	52.52	20.06
$S_{d,4\;\mathrm{MHz}}$ in kPa	127	210	80

**Table 3:** Overview of the sensor size and surface tilt sensing ranges for the single speckle sensing approach.

	Single Speckle Analysis
$d_{px}$ in μm	1600
$x/y_{sp,max}$ in μm	1120
$\alpha_{max,x/y}$ in ${}^{\circ }$	0.0642

## Data Availability

The data that supports the findings of the study are provided within the article.

## Abbreviations

The following abbreviations are used in this manuscript:

APS	avalanche-photodiode sensor
MPE	maximum permitted exposure
PA	Photoacoustic
PhAT	phantom Setup A transmission mode
PhAR	phantom Setup A reflection mode
PhBT	phantom Setup B transmission mode
PVCP	polyvinyl chloride plastisol
skinAT1	skin sample Setup A transmission mode
skinAT2p	skin sample Setup A transmission mode pulsed illumination
